# Multidisciplinary physician survey assessing knowledge of the female athlete triad and relative energy deficiency in sport

**DOI:** 10.1186/s40337-023-00800-4

**Published:** 2023-05-09

**Authors:** Alexandra E. Warrick, Brandon Hassid, Brandon Coleman, Catherine Cansino, Marcia Faustin

**Affiliations:** 1 Department of Family & Community Medicine, University of California Davis School of Medicine, 4860 Y street Suite 3850, Sacramento, CA 95816 USA; 2 Department of Physical Medicine and Rehabilitation, University of California Davis School of Medicine, 4860 Y Street, Suite 3850, Sacramento, CA 95817 USA; 3 Department of Obstetrics and Gynecology, University of California Davis School of Medicine, 2521 Stockton Blvd, 4th floor, Sacramento, CA 95817 USA

**Keywords:** Female, Athlete, Triad, Relative, Energy deficiency in sport, Low energy availability, RED-S

## Abstract

**Background:**

Short and long-term health consequences surrounding Low Energy Availability can be mitigated by recognizing the risk factors and making early diagnosis of the Female Athlete Triad (Triad) and Relative Energy Deficiency in Sport (RED-S). While awareness of the Triad among physicians and allied health professionals has been studied, there are very few studies that assess physician awareness of both the Triad and RED-S.

**Methods:**

Our study assesses Low Energy Availability, the Triad, and RED-S knowledge with an electronic survey, educational handout, and follow up survey among physicians across multiple specialties at a single academic institution.

**Results:**

Among 161 respondents, respective Triad and RED-S awareness among surveyed specialties was highest in Orthopedic surgeons (100%, 100%), followed by Physical Medicine & Rehabilitation (70%, 53%), Family Medicine (67%, 48%), Internal Medicine (54%, 36%), Obstetrics and Gynecology (46%, 32%), Pediatrics (45%, 29%), Endocrinology (33%, 33%), and Other (33%, 33%). Comparing the initial survey results to the follow-up survey results, there was an increase from 37 to 72% of physicians who correctly identified that the presence of low BMI or recent weight loss is not a required component of the Triad or RED-S. Both the initial and follow-up survey revealed a continued misperception surrounding the use of hormonal contraception to resume menstrual cycles, with 33% of physicians on initial survey and 44% of physicians on follow-up survey incorrectly answering that question.

**Conclusions:**

Multidisciplinary physicians have various levels of knowledge surrounding the Triad and RED-S, and there is a need for improved physician awareness, diagnosis, and treatment of the Triad and RED-S. Misperceptions exist surrounding the role of hormonal contraception in female athletes with the Triad and RED-S to regain and regulate menses.

**Supplementary Information:**

The online version contains supplementary material available at 10.1186/s40337-023-00800-4.

## Abstract (summary)

When there is insufficient energy intake compared to energy output, leading to low energy availability, this lack of energy can strain the body’s metabolic needs. The Female Athlete Triad (Triad) and/or Relative Energy Deficiency in Sport (RED-S) refer to this strain that affects organ systems. While these syndromes exist, it is unclear if there is a differential in awareness among physicians from different specialties. Our study confirms that physician awareness varied among different specialties, and misperceptions continue to exist surrounding hormonal contraception and regulating menses.

**Supplementary Information:**

The online version contains supplementary material available at 10.1186/s40337-023-00800-4.

## Background

The Female Athlete Triad (Triad) and Relative Energy Deficiency in Sport (RED-S) overlap by the presence of low Energy Availability (EA) that leads to negative health consequences [1, 2]. Low EA is defined by insufficient energy intake relative to energy expenditure [1, 3, 4] (See Additional file 1: for EA calculation).

The Triad, originally defined in 1992 and later redefined in 2007, is a spectrum of “low EA with or without disordered eating, menstrual dysfunction, and low bone mineral density among female athletes.”[1, 6, 7] RED-S was introduced in 2014 by the International Olympic Committee (IOC) as a complex clinical syndrome that expands upon the Triad criteria to include men as well as multiple organ system health and performance consequences related to impaired physiological functioning [2, 4, 5, 8].

Low EA can have a clinical spectrum of serious short- and long-term bone and reproductive health consequences, the most severe being amenorrhea, osteoporosis, and hypogonadotropic hypogonadism [1, 9, 10]. There is a need for continuous patient assessment and increased educational efforts to support awareness, diagnosis, and treatment of low EA in all individuals [11, 12].

A study comparing the prevalence of two Triad components among elite female athletes (5.4–26.9%) to recreational athletes (12.4–15.2%) revealed that low EA can occur across a variety of activity levels and sports [13, 14]; it can impact elite, endurance, lean sport, and recreational athletes [3, 14]. Physicians from various specialties will encounter patients with low EA who experience varying symptoms, and a high level of clinical suspicion is necessary to avoid a missed diagnosis and treatment opportunity. Given the varied and, at times, subtle presentation of low EA, recognizing the symptoms of low EA remains challenging for medical professionals, allied health professionals, parents/guardians, and athletes themselves [11, 12, 15–21].

Previous surveys have demonstrated the need for increased physician awareness of the Triad and RED-S. 47% of physicians surveyed across three large academic institutions recognized the hallmark Triad features [11]. While the Triad has been acknowledged for many years, RED-S was defined less than a decade ago [2]. In 2020, a survey revealed that 36% of physicians had knowledge of RED-S compared to 24% of physician assistants, physical therapists, nurse practitioners, certified athletic trainers, and trainees [12]. Physicians trained in Sports Medicine were most aware of the Triad (91%) and RED-S (61%), while only 13% of physicians and allied health professionals felt comfortable treating patients with RED-S [12]. While our survey focuses on knowledge surrounding the Female Athlete Triad and RED-S, we recognize the Male Athlete Triad as a separate related diagnosis but did not directly address it with this study [9, 22, 23].

The aim of our study is to assess the awareness of both the Triad and RED-S among multidisciplinary physicians, and to determine if an educational handout can improve awareness of the Triad and RED-S on a follow up survey. Very few studies have separately assessed awareness of the Triad or RED-S among various healthcare providers [12, 18]. To our knowledge, no prior studies have simultaneously assessed awareness of both the Triad and RED-S among multidisciplinary physicians, and no prior studies have assessed knowledge of these topics after an educational intervention. For our survey, we hypothesize that the majority of physicians surveyed are not aware of the key features of the Triad and RED-S, and that providing educational materials will increase their knowledge base.

## Methods

We distributed electronic surveys at University of California (UC), Davis, an academic, tertiary medical center in Northern California. With approval from the UC Davis Biomedical Institutional Review Board, we surveyed attending, fellow, and resident physicians from Emergency Medicine, Endocrinology, Family Medicine, Internal Medicine, Orthopedics, Obstetrics & Gynecology (OBGYN), Physical Medicine & Rehabilitation (PM&R), Pediatrics, and Psychiatry. Department administrators electronically mailed the surveys to their respective listservs of eligible participants for the study. The initial survey was distributed in April 2021 and the follow up survey was sent out in May 2021, approximately five weeks after the initial survey.

In developing the survey, questions were reviewed by several physicians at UC Davis and other academic institutions, and responses were considered correct based on the most recent Triad and RED-S consensus statements [1, 2, 10]. The UC Davis physicians who reviewed the questions were excluded from study participation. The physicians that reviewed the surveys included physicians from multidisciplinary backgrounds including, Family Medicine, Internal Medicine, Physical Medicine & Rehabilitation and Primary Care Sports Medicine. Qualtrics (R) (Provo, UT), a web-based analytic software platform, was used to administer the surveys. De-identified participant data with unique identifiers were saved in a HIPPA-compliant manner. Initial and follow-up survey responses were linked using a unique identifier.

The initial survey included 18 multiple-choice questions (including demographic data, see Additional file 1), and the follow up survey included 12 multiple-choice questions (see Additional file 1). Survey respondents were encouraged, but not required, to complete all questions. The survey did not require respondents to respond to all questions on either survey, leading to a variable response rate among questions. For questions that allowed more than one response, the response was categorized as correct if all correct variables were selected; partial credit was not awarded. Survey duration was estimated to range from three to five minutes. Two email reminders were sent for each survey, encouraging participation and incentivizing eligible survey participants with entry into a raffle to earn one of two $50 Amazon gift cards. Respondents received the correct answers and an educational handout immediately after completing the initial survey. This two-page educational handout, developed by this research team, summarizes key elements in recognizing, diagnosing, and treating athletes with low EA (See Additional file 1).

### Statistical methods

Statistical analyses were performed using SAS 9.4 software (Cary, NC). We used McNemar’s tests to analyze paired responses from initial and follow-up survey results. A *p*-value less than 0.05 was considered statistically significant.

## Results

Among 834 potential participants, 161 physicians (19%) completed the initial survey. There were no responses from the Emergency Medicine and Psychiatric department (*n* = 265), and if they were removed from total potential participants, the total response rate would increase to 28%. Of those 161 physicians, 49 (30.4%) completed the follow up survey. The specialties with the highest response rate for the initial survey were Physical Medicine & Rehabilitation (70%) & Obstetrics/Gynecology (51%). The remaining specialties were under 40% (in descending order: Family Medicine, Pediatrics, Internal Medicine/Endocrinology, Orthopedics, Emergency Medicine, and Psychiatry). There were similar demographic characteristics of respondents between the initial and follow-up surveys (Table 1). The initial and follow-up survey results were only compared if the participant responded to the corresponding questions in both surveys, as some participants submitted surveys with partial responses. There were fewer responses on some questions compared to others. There was a 70% decrease in participation from the initial survey (*n* = 161) to the follow-up survey (*n* = 49). Additionally, some physicians answered the survey several times with incomplete results. Duplicate entries were removed.

**Table 1:** Demographics

Profession	
Attending physician	93 (59%)
Fellow physician	5 (3%)
Resident physician	60 (38%)
Sports fellowship	
Yes	8 (5%)
No	149 (94%)
Not listed	1 (1%)

While 161 physicians participated in the survey, only 158 physicians provided demographic data

Among the physicians who participated in the initial survey (*n* = 161), 48% correctly identified the three criteria of the Triad, whereas 12% did not answer this question and 40% answered incorrectly. In comparison, among physicians who completed both the initial and follow-up surveys (*n* = 46), 54% correctly identified the three Triad criteria. For reference, the Triad criteria survey question is below. Correct answers are marked in bold.

**Table:** 

**Survey Question—Triad** What are the three criteria for the ‘Female Athlete Triad’ (Triad)? Instructions: Please check the three that apply ** a. Menstrual dysfunction** ** b. Low energy availability with or without disordered eating** c. Weight loss > 10lbs d. Taking hormonal contraception **e. Lower than expected bone mineral density ± stress fractures**	

**Table:** 

**Survey Question – RED-S** What are the features of RED-S? Please check all that apply ** a. Term that encompasses low energy availability, including components of the Female Athlete Triad** ** b. Syndrome that affects physiologic function, health, and athletic Performance** ** c. Only endurance athletes are affected** ** d. Male and female athletes are affected** ** e. Includes able-bodied and disabled athletes**	

Of the physicians who participated in the initial survey (*n* = 161), 32% correctly identified the key RED-S features, whereas 18% did not answer this question and 50% answered incorrectly. Of the respondents who answered both surveys (*n* = 49), 40% correctly identified features of RED-S. From the initial to the follow-up survey, the percentage of correct RED-S recognition (Survey Question – RED-S) improved from 40 to 63% (*p* = 0.0129) among those who answered both surveys (*n* = 43).

Comparing the initial survey to the follow-up survey, the percentage of correct Triad recognition (Survey Question – Triad) improved from 54 to 70% (*p* = 0.0654) among those who answered both surveys (*n* = 46). Physicians who answered incorrectly on the initial survey were more likely to answer correctly on the follow-up survey as compared to random guessing (*p* < 0.0001).

**Fig. 1:**
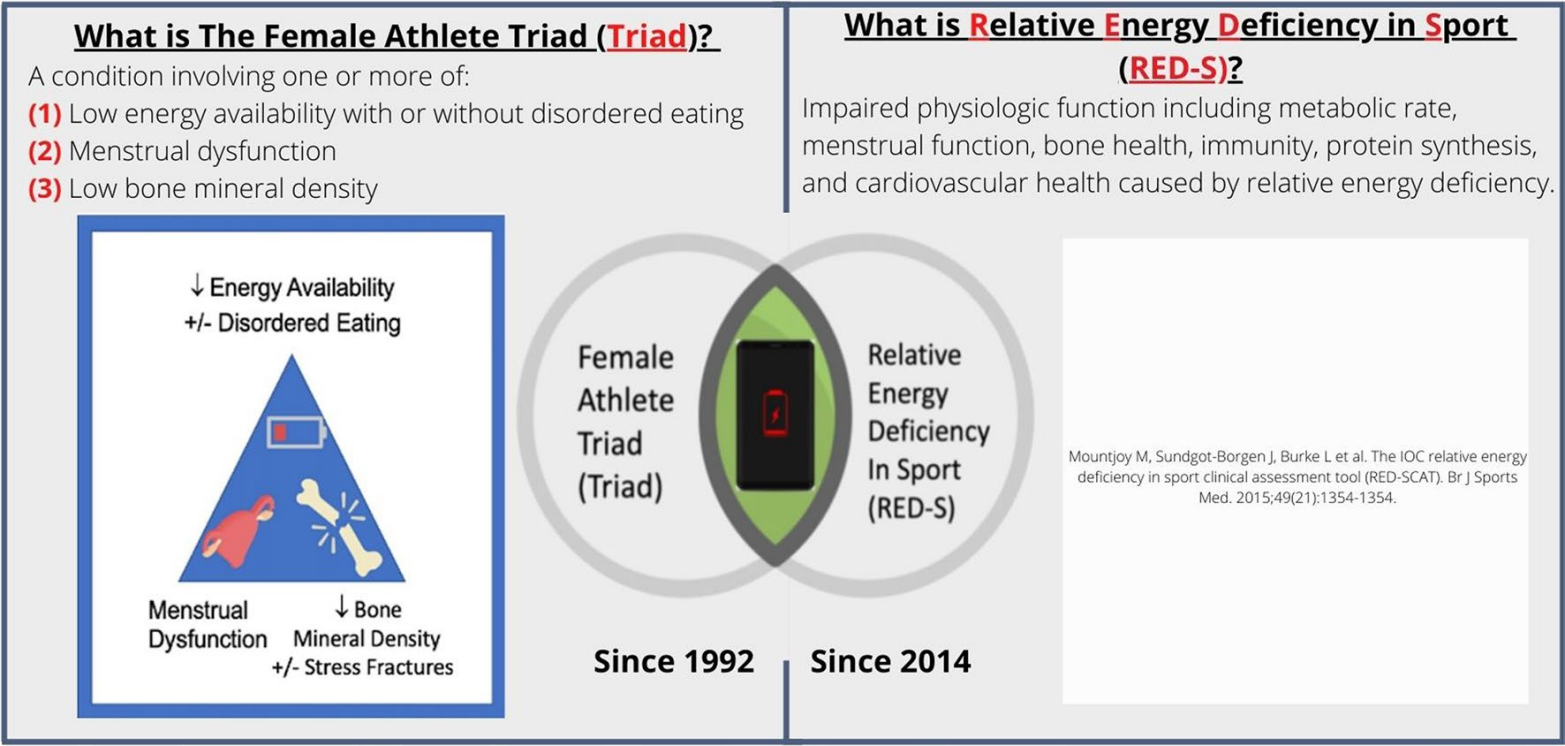
Triad and RED-S Comparison—Educational Handout. Reference from our Educational Handout (See Additional file 1) [1, 4, 5]

**Fig. 2:**
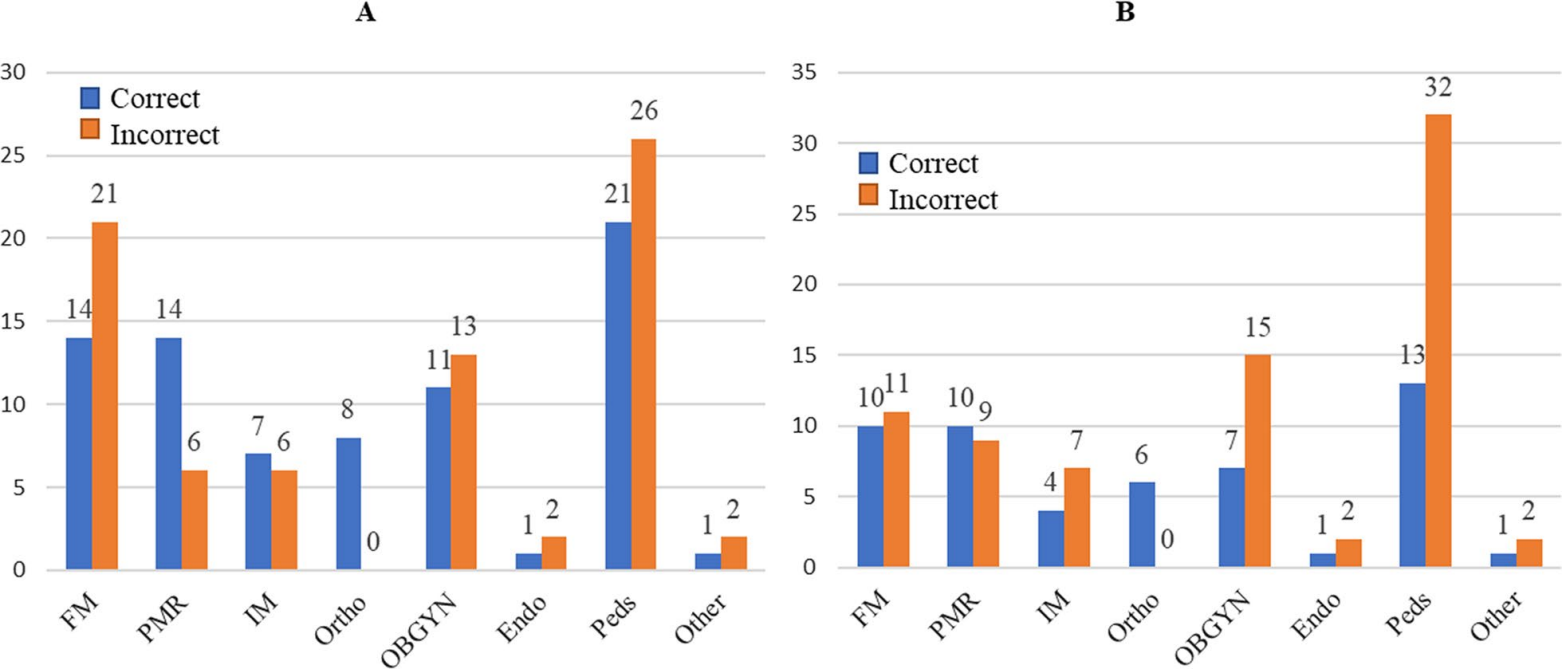
**A.** Number of respondents by specially who correctly and incorrectly identified the criteria for the female athlete triad. **B**. Number of respondents by specially who correctly and incorrectly identified the criteria for the relative energy deficiency in sport

Based on medical specialty, there was a broad range of Triad awareness (Fig. 1A and 1B) and RED-S awareness (Fig. 2A and 2B). Recognition was greatest among Orthopedic Surgery for both the Triad and RED-S. Based on medical specialty, there was a broad range of Triad and RED-S awareness. Despite a small sample size, (Fig. 2A and 2B), recognition was greatest among Orthopedic Surgery for both the Triad and RED-S. The specialty with the highest percentage to correctly identify Female Athlete Triad was Orthopedics (100%), followed by Physical Medicine & Rehabilitation (70%), Family Medicine (67%), Internal Medicine (54%) Obstetrics and Gynecology (46%), Pediatrics (45%), Endocrinology (33%), and ‘Other’ who did not choose to provide their training background/specialty (33%). The specialty with the highest percentage to correctly identify RED-S was Orthopedics (100%), followed by Physical Medicine & Rehabilitation (53%), Family Medicine (48%), Internal Medicine (36%), Obstetrics and Gynecology (32%), Endocrinology (33%), Other (33%), and Pediatrics (29%). See Fig. 2A and 2B for number of survey respondents per specialty, including the number of correct and incorrect responses.

### Addressing misperceptions

Four questions were aimed to address Low EA/Triad/RED-S misperceptions. Data described are reflective of those who answered these questions on both surveys.

**Table:** 

**1**^**st**^** Question:** “Is the presence of low BMI or recent weight loss a required component of the Triad or RED-S?” Yes/No	

Correct response - No

Among physicians who answered both surveys (*n*=46), 37% answered correctly on the initial survey. Low BMI or recent weight loss are not required criteria, and some people may present with low energy availability despite a normal or elevated BMI, or no change in weight. Comparing the initial survey to the follow up survey, correct responses improved from 37 to 72% (*p* = 0.0001).

**Table:** 

**2**^**nd**^** Question:** “Can Low Energy Availability be present without a change in weight?” Yes/No	

Correct response – Yes

100% of physicians answered this correctly. A change in weight is not needed to be diagnosed with low EA.

**Table:** 

**3**^**rd**^** Question:** “Menstrual dysfunction/abnormal uterine bleeding is a risk factor for poor bone health.” True/False	

Correct response – True

91% of physicians correctly recognized that this is a risk factor for poor bone health. Correct responses improved from 91 to 100% from the initial to follow up survey.

**Table:** 

**4**^**th**^** Question:** “Hormonal contraception is recommended in athletes with the Triad/RED-S to resume menses.” True/False	

Correct response – False

67% responded correctly with “false.” Further workup is needed, and prescribing contraception may mask underlying negative health impacts from low energy availability. While most results comparing the follow up survey to the initial survey demonstrate an improvement in knowledge, for this question there was a decrease in correct answers among 45 respondents from 67% to 56% (*p* = 0.2668) surrounding the use of hormonal contraception to resume menses.

## Discussion

Overall, there is a need to educate physicians about low EA, the Triad, and RED-S. This survey study demonstrates the relatively low awareness of the Triad and RED-S among many specialties that have the opportunity to identify and potentially screen for these syndromes. Similar to the findings from Curry et al. [11], orthopedic surgeons are more likely to accurately identify the Triad and RED-S criteria compared to other specialties. We hypothesized that the majority of physicians surveyed were not aware of Triad and RED-S criteria, and that providing educational materials would increase their knowledge base. Fewer physicians correctly identified RED-S compared to the Triad among physicians who completed both surveys, likely because RED-S is a more recently defined syndrome with less body of evidence than the Triad. There was an improvement in ability to correctly identify Triad and RED-S criteria from the initial to the follow-up survey. However, we cannot state that our handout was the direct cause of this improvement.

We acknowledge the limitations of our study including the small sample size and large decrease in participation from the initial to the follow-up survey, leading to challenges in drawing conclusions about changes from the initial to the follow up survey. Survey participants were not required to answer all questions and therefore, survey results may be biased. Other limitations included only providing full credit for recognition of all components on the multiple-choice type of questions and answers. This likely undervalues the knowledge that may have been detected by assessing partially correct responses. Also, multiple-choice questions can be interpreted in different ways and lead to variable answers.

Interestingly, our initial survey response rate of all potential participants of 19%, similar to the 23.5% response rate of the survey regarding physician awareness of Triad criteria by Curry et. al across three large academic hospitals [11]. The low response rate is reflective of a common challenge with emailed survey questions, with decreasing response rate with each subsequent survey request. [26, 27] There were no participants who identified as Emergency Medicine nor Psychiatry physicians, which may be attributable to not receiving the survey despite multiple reminders to administrators.

## Conclusion

A multidisciplinary approach to diagnose and treat low EA syndromes, the Triad, and RED-S is of utmost importance. Our study demonstrates a need to educate physicians from multiple specialties regarding these syndromes. Early recognition can prompt treatment and protect athletes’ short- and long-term health.

Please see our educational handout in the Additional file 1, as well as current educational resources available online, and share with colleagues.

Additional resources for reference include:

- Monitoring and Return to Play: RED-SCAT [5]:
  o https://bjsm.bmj.com/content/bjsports/49/7/421.full.pdf
- Female Athlete Triad Consensus Statement [10]:
  o https://bjsm.bmj.com/content/48/4/289
- RED-S IOC Consensus Statement [2]:
  o https://bjsm.bmj.com/content/48/7/491
- Downloadable Sports Nutrition Handouts for Patient Education: [24]
  o https://www.sportsrd.org/downloadable-resources/

## Supplementary Information

**Additional file 1.** Triad and RED-S Comparison—Educational Handout.

## Data Availability

The datasets used and/or analyzed during the current study are available from the corresponding author on reasonable request.

## References

[1] Matzkin E, Curry EJ, Whitlock K (2015). Female athlete triad: past, present, and future. JAAOS-J Am Acad Orthopaedic Surg.

[2] Mountjoy M, Sundgot-Borgen J, Burke L, Carter S, Constantini N, Lebrun C (2014). The IOC consensus statement: beyond the female athlete triad-relative energy deficiency in sport (RED-S). Br J Sports Med.

[3] Loucks AB (2007). Low energy availability in the marathon and other endurance sports. Sports Med.

[4] Mountjoy M, Sundgot-Borgen JK, Burke LM, Ackerman KE, Blauwet C, Constantini N (2018). IOC consensus statement on relative energy deficiency in sport (RED-S): 2018 update. Br J Sports Med.

[5] Mountjoy M, Sundgot-Borgen J, Burke L, Carter S, Constantini N, Lebrun C (2015). The IOC relative energy deficiency in sport clinical assessment tool (RED-S CAT). Br J Sports Med.

[6] Otis CL, Drinkwater B, Johnson M, Loucks A, Wilmore J (1997). American college of sports medicine position stand the female athlete triad. Med Sci Sports Exercise.

[7] Yeager KK, Agostini R, Nattiv A, Drinkwater B (1993). The female athlete triad: disordered eating, amenorrhea, osteoporosis. Med Sci Sports Exerc.

[8] Ackerman KE, Holtzman B, Cooper KM, Flynn EF, Bruinvels G, Tenforde AS (2019). Low energy availability surrogates correlate with health and performance consequences of relative energy deficiency in sport. Br J Sports Med.

[9] Nattiv A, De Souza MJ, Koltun KJ, Misra M, Kussman A, Williams NI (2021). The male athlete triad-a consensus statement from the female and male athlete triad coalition part 1: definition and scientific basis. Clin J Sport Med.

[10] De Souza MJ, Nattiv A, Joy E, Misra M, Williams NI, Mallinson RJ (2014). 2014 female athlete triad coalition consensus statement on treatment and return to play of the female athlete triad: 1st international conference held in San Francisco, California, may 2012 and 2nd international conference held in Indianapolis, Indiana, May 2013. Br J Sports Med.

[11] Curry EJ, Logan C, Ackerman K, McInnis KC, Matzkin EG (2015). Female athlete triad awareness among multispecialty physicians. Sports Med Open.

[12] Tenforde AS, Beauchesne AR, Borg-Stein J, Hollander K, McInnis K, Kotler D (2020). Awareness and comfort treating the female athlete triad and relative energy deficiency in sport among healthcare providers. Dtsch Z Sportmed.

[13] Gibbs JC, Williams NI, De Souza MJ (2013). Prevalence of individual and combined components of the female athlete triad. Med Sci Sports Exerc.

[14] Torstveit MK, Sundgot-Borgen J (2005). The female athlete triad: are elite athletes at increased risk?. Med Sci Sports Exerc.

[15] Pantano KJ (2006). Current knowledge, perceptions, and interventions used by collegiate coaches in the US. Regarding the prevention and treatment of the female athlete triad. N Am J Sports Phys Ther.

[16] Brown KN, Wengreen HJ, Beals KA (2014). Knowledge of the female athlete triad, and prevalence of triad risk factors among female high school athletes and their coaches. J Pediatr Adolesc Gynecol.

[17] Kroshus E, Sherman RT, Thompson RA, Sossin K, Austin SB (2014). Gender differences in high school coaches’ knowledge, attitudes, and communication about the female athlete triad. Eat Disord.

[18] Kroshus E, DeFreese JD, Kerr ZY (2018). Collegiate athletic trainers’ knowledge of the female athlete triad and relative energy deficiency in sport. J Athl Train.

[19] Warrick A, Faustin M, Waite B (2020). Comparison of female athlete triad (triad) and relative energy deficiency in sport (RED-S): a review of low energy availability, multidisciplinary awareness, screening tools and education. Curr Phys Med Rehabil Rep.

[20] Javed A, Tebben PJ, Fischer PR, Lteif AN (2013). Female athlete triad and its components: toward improved screening and management. Mayo Clin Proc.

[21] Bonci CM, Bonci LJ, Granger LR, Johnson CL, Malina RM, Milne LW (2008). National athletic trainers’ association position statement: preventing, detecting, and managing disordered eating in athletes. J Athl Train.

[22] Fredericson M, Kussman A, Misra M, Barrack MT, De Souza MJ, Kraus E (2021). The male athlete triad-a consensus statement from the female and male athlete triad coalition part II: diagnosis, treatment, and return-to-play. Clin J Sport Med.

[23] Tenforde AS, Barrack MT, Nattiv A, Fredericson M (2016). Parallels with the female athlete triad in male athletes. Sports Med.

[24] Downloadable resources - CPSDA | SportsRd.org | collegiate & professional sports dietitians association [Internet]. [cited 2022 Sep 23]. Available from: https://sportsrd.org/downloadable-resources/

[25] Loucks AB, Kiens B, Wright HH (2011). Energy availability in athletes. J Sports Sci.

[26] Cook DA, Wittich CM, Daniels WL, West CP, Harris AM, Beebe TJ (2016). Incentive and reminder strategies to improve response rate for internet-based physician surveys: a randomized experiment. J Med Internet Res.

[27] Barnhart BJ, Reddy SG, Arnold GK (2021). Remind me again: physician response to web surveys: the effect of email reminders across 11 opinion survey efforts at the American board of internal medicine from 2017 to 2019. Eval Health Prof.

