# Supplementary material for: Multidisciplinary physician survey assessing knowledge of the female athlete triad and relative energy deficiency in sport
Source: J Eat Disord. 2023 May 9;11:70. doi: 10.1186/s40337-023-00800-4 (PMC10169360; doi:10.1186/s40337-023-00800-4)
Supplement: Supplementary file 1 — Additional file 1. Triad and RED-S Comparison—Educational Handout. [file 40337_2023_800_MOESM1_ESM.docx]

Supplementary Material

**Educational Handout**


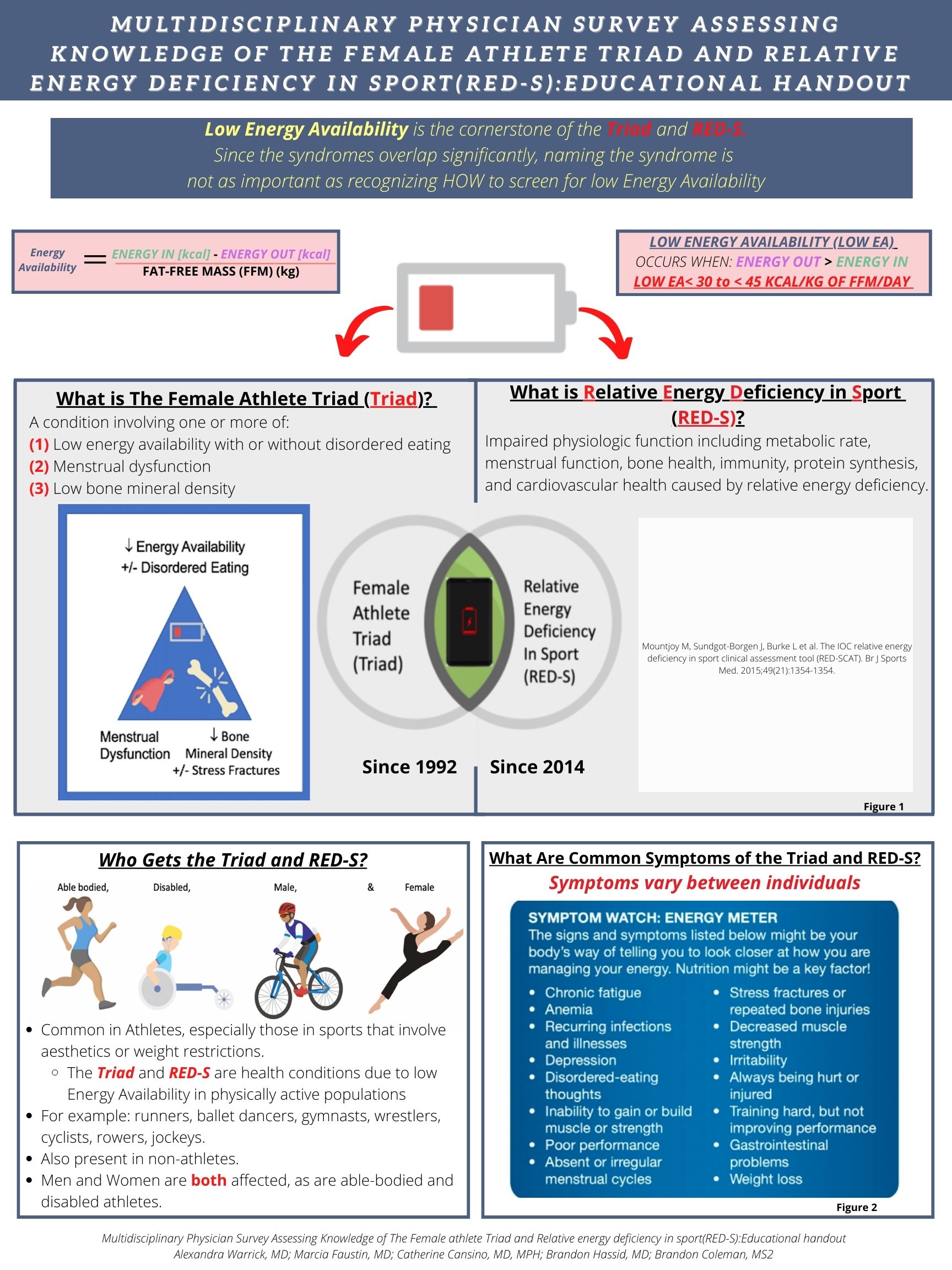


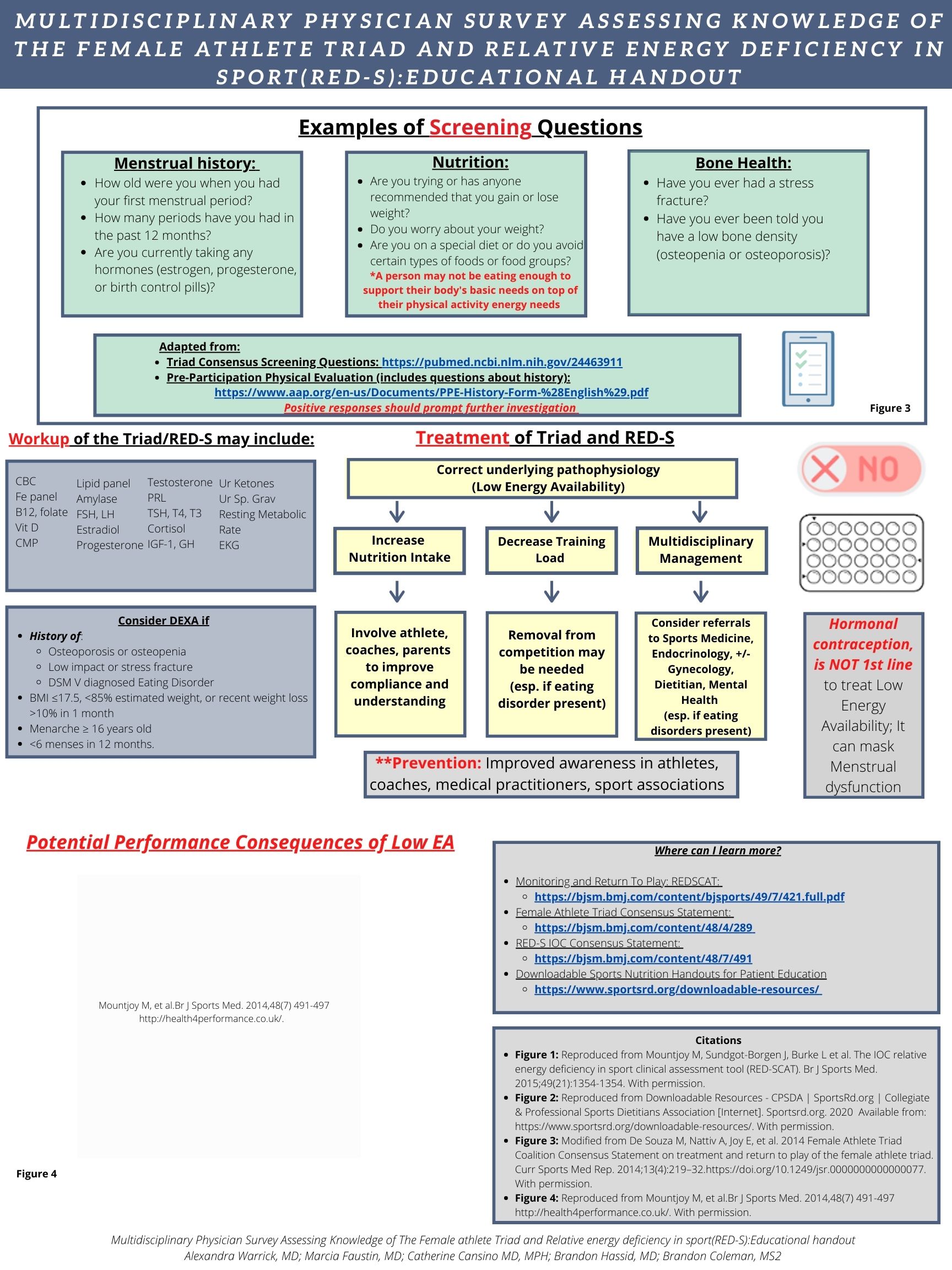


**Initial Survey:**

| **1. What is your current level of training?**   1. Resident 2. Fellow 3. Attending     **2. What is your specialty from residency training?**   1. Family Medicine 2. Physical Medicine & Rehabilitation 3. Internal Medicine 4. Orthopedic Surgery 5. Obstetrics & Gynecology 6. Psychiatry 7. Endocrinology 8. Pediatrics 9. Emergency Medicine 10. Other – write in     **3. Have you completed a Sports Medicine Fellowship?**   1. Yes 2. No     **4. What age range are your patients? Please select all that apply.**   1. ≤ 10 years old 2. 10 – 15 years old 3. 15 – 30 years old 4. 31 – 45 years old 5. 46 – 60 years old 6. 60 + years old 7. All of the above     **5. How familiar are you with the diagnosis of Female Athlete Triad (Triad) on a scale of 1 to 5?**   1. 1 – not familiar 2. 2 3. 3 4. 4 5. 5 – well versed in the diagnosis and treatment     **6. How familiar are you with the diagnosis of Relative Energy Deficiency in Sport’ (RED-S) on a scale of 1 to 5?**   1. 1 – not familiar 2. 2 3. 3 4. 4 5. 5 – well versed in the diagnosis   **7. What is your level of comfort in screening for the Triad and RED-S on a scale from 1 to 5?**   1. 1 – not comfortable 2. 2 3. 3 4. 4 5. 5 - very comfortable   **8. What are the three criteria for the ‘Female Athlete Triad’ (Triad)? Please check the three that apply.**   1. **Menstrual dysfunction** 2. **Low energy availability with or without disordered eating** 3. Weight loss > 10lbs 4. Taking hormonal contraception 5. **Lower than expected bone mineral density +/- stress fractures**     **9. What are the features of RED-S? Please check all that apply.**   1. **Term that encompasses low energy availability, including components of the Female Athlete Triad** 2. **Syndrome that affects physiologic function, health, and athletic Performance** 3. Only endurance athletes are affected 4. **Male and female athletes are affected** 5. **Includes able-bodied and disabled athletes**     **10. Of the** **sports listed, which 3 sports put athletes at most risk of the Triad/RED-S?**  a. Softball, Baseball, Basketball  **b.** **Running, Ballet dancing, Wrestling**  c. Roller derby, Golf, Bowling  d. Tennis, Hockey, Football    **11. Is the presence of low BMI or recent weight loss a required component of the Triad or RED-S?**  a. Yes  **b.** **No**  c. I don’t know      **12. Can Low Energy Availability be present without a change in weight?**   1. **Yes** 2. No       **13. Menstrual dysfunction/abnormal uterine bleeding is a risk factor for low bone mineral density +/- stress fractures.**  **a. True**  b. False    **14. Hormonal contraception is recommended in athletes with the Triad/RED-S to resume menses.**  a. True  **b.** **False**    **15. Which of the following may be part of the initial medical workup for the Triad/RED-S.**  **Please check all that apply.**  **a.** **CBC**  **b.** **Iron panel**  **c.** **TSH, Free T4, T3**  **d.** **FSH, LH**  e. Copper  **f.** Aldolase    **16. Which** **of the following tools do you use in your practice to screen for the Triad/RED-S?**  **Please check all that apply.**  a. History  b. Questionnaires  a. Periodic Health Exam  b. Pre-participation exam (PPE)  c. Low Energy Availability in Females Questionnaire (LEAF-Q)  d. RED-S screening tools  e. Triad Risk Assessment Tool  f. Eating Disorder/Disordered Eating screening tools  g. Other questionnaires  c. Diet log  d. Athletic Training log  e. Activity tracker  f. Weight changes  g. Other – write in  h. None of the above      **17. If you had a patient exhibiting symptoms of low energy availability, how do you manage with work up and treatment plan?**   1. No referral, manage them on my own   **b.** Initiate diagnostic work up and refer to specialist  **c.** Refer to specialist immediately.  **d.** Other, please specify below.    **18. If your patient presents with low energy availability and symptoms/signs of the Triad/RED-S, which specialists may potentially be considered for referral?**  **a.** Sports Medicine  **b.** Psychiatry (Psychiatrist, Psychologist)  **c.** Sports Dietitian  **d.** Endocrinology  **e.** Obstetrics/Gynecology  **f.** Orthopedic Surgeon    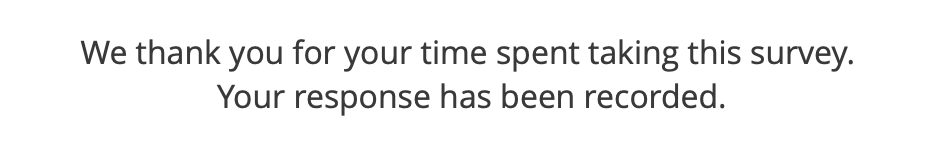 |
| --- |

**Follow Up Survey:**

| 1. After taking the survey I sought additional knowledge regarding the Triad/RED-S:   a. True  b. False     1. I am more familiar with screening methods for the triad after taking the survey and reading the handout.   a. True  b. False     1. How has the survey and handout impacted your clinical practice? Please select all that apply.   a. Increased knowledge about the Triad  b. Increased knowledge about RED-S  c. Increased knowledge of screening questions  d. Increased knowledge of screening tools/resources  e. Increased knowledge about the diagnostic workup  f. Increased use of screening tools/resources  g. All of the above.  f. Other, write in:     1. Please provide any additional feedback for how you would like to receive additional information on the topic: write in      1. Please provide any feedback: write in        1. **What are the three criteria for the ‘Female Athlete Triad’ (Triad)?**   **Please check the three that apply.**  **a. Menstrual dysfunction**  **b. Low energy availability with or without disordered eating**  c. Weight loss > 10lbs  d. Taking hormonal contraception  **e. Lower than expected bone mineral density +/- stress fractures**     1. **What are the features of Relative Energy Deficiency in Sport (RED-S)?**   **Please check all that apply.**  **a. Term that encompasses low energy availability, including components**  **of the Female Athlete Triad**  **b. Syndrome that affects physiologic function, health, and athletic**  **performance**  c. Only endurance athletes are affected  **d. Male and female athletes are affected**  **e. Includes able-bodied and disabled athletes**       1. **Of the** **sports listed, which 3 sports put athletes at most risk of the Triad/RED-S?**   a. Softball, Baseball, Basketball  **b.** **Running, Ballet dancing, Wrestling**  c. Roller derby, Golf, Bowling  d. Tennis, Hockey, Football     1. **Is the presence of low BMI or recent weight loss a required component of the Triad or RED-S?**   a. Yes  **b.** **No**  c. I don’t know     1. **Can Low Energy Availability be present without a change in weight?**   **a.** **Yes**  b. No     1. **Menstrual dysfunction/abnormal uterine bleeding is a risk factor for low bone mineral density +/- stress fractures.**   **a. True**  b. False     1. **Hormonal contraception is recommended in athletes with the Triad/RED-S to resume menses.**   a. True  **b.** **False** |
| --- |
|  |

________________________________________________________________________

**Energy Availability Calculation:** [[25]](https://sciwheel.com/work/citation?ids=12698569&pre=&suf=&sa=0&dbf=0)

Energy Availability (EA) is defined as dietary energy intake [kcal] minus the energy expended in exercise [kcal], divided by Fat-Free Mass [kg], with units kcal/kg FFM/day.

| $EA=\frac{EI-EEE}{FFM}$ kcal/kg FFM/day | EA = Energy Availability  EI= Energy Intake  EEE=Energy expended in exercise  FFM= Fat-Free Mass |
| --- | --- |
